# X-ray Diffraction Studies on the Structural Origin of Dynamic Tension Recovery Following Ramp-Shaped Releases in High-Ca Rigor Muscle Fibers

**DOI:** 10.3390/ijms21041244

**Published:** 2020-02-13

**Authors:** Haruo Sugi, Maki Yamaguchi, Tetsuo Ohno, Hiroshi Okuyama, Naoto Yagi

**Affiliations:** 1 Department of Physioloogy, Teikyo University School of Medicine, Tokyo 173-8605, Japan; 2 Department of Molecular Physiology, Jikei University School of Medicine, Tokyo 105-0003, Japan; maki@jikei.ac.jp (M.Y.); okuyamah@wb3.so-net.ne.jp (H.O.); 3 Department of Sports Medicine, Teikyo Heisei University, Chibaken 290-0193, Japan; t269ohno@docomo.ne.jp; 4 SPring-8, Hyogoken 675-5198, Japan; Yagi@spring8.or.jp

**Keywords:** muscle contraction, rigor muscle fiber, myosin head tilting, X-ray diffraction, myofilament lattice

## Abstract

It is generally believed that during muscle contraction, myosin heads (M) extending from myosin filament attaches to actin filaments (A) to perform power stroke, associated with the reaction, A-M-ADP-Pi → A-M + ADP + Pi, so that myosin heads pass through the state of A-M, i.e., rigor A-M complex. We have, however, recently found that: (1) an antibody to myosin head, completely covering actin-binding sites in myosin head, has no effect on Ca^2+^-activated tension in skinned muscle fibers; (2) skinned fibers exhibit distinct tension recovery following ramp-shaped releases (amplitude, 0.5% of Lo; complete in 5 ms); and (3) EDTA, chelating Mg ions, eliminate the tension recovery in low-Ca rigor fibers but not in high-Ca rigor fibers. These results suggest that A-M-ADP myosin heads in high-Ca rigor fibers have dynamic properties to produce the tension recovery following ramp-shaped releases, and that myosin heads do not pass through rigor A-M complex configuration during muscle contraction. To obtain information about the structural changes in A-M-ADP myosin heads during the tension recovery, we performed X-ray diffraction studies on high-Ca rigor skinned fibers subjected to ramp-shaped releases. X-ray diffraction patterns of the fibers were recorded before and after application of ramp-shaped releases. The results obtained indicate that during the initial drop in rigor tension coincident with the applied release, rigor myosin heads take up applied displacement by tilting from oblique to perpendicular configuration to myofilaments, and after the release myosin heads appear to rotate around the helical structure of actin filaments to produce the tension recovery.

## 1. Introduction

Muscle contraction is caused by relative sliding between actin and myosin filaments coupled with ATP hydrolysis [1,2], which in turn is produced by attachment-detachment cycles between myosin heads extending from myosin filaments and the corresponding myosin-binding sites in actin filaments. Based on biochemical studies on the ATPase reaction steps of actomyosin in solution [3], myosin head (M) first attaches to actin filaments (A) in the form of M-ADP-Pi, and performs a power stroke associated with the reaction, A-M-ADP-Pi → A-M + ADP + Pi, so that at the end of power stroke myosin head takes the form of A-M, which is known as rigor A-M complex. Upon binding with a next ATP, M detaches from A to perform a recovery stroke associated with reaction, A-M + ATP → A + M-ATP → A + M-ADP-Pi, and then again attaches to A. It is generally believed that the A-M in the above biochemical scheme corresponds to a high-affinity complex formed in the absence of ATP, i.e., rigor A-M linkages in rigor muscle. The structure of the rigor A-M complex has been studied electron microscopically using extracted protein samples [4,5]. Based on the static nature of rigor linkages, it is implicitly believed that tension is passively maintained in rigor muscle.

Contrary to the above general view, however, we have found that neither Ca^2+^-activated muscle fiber contraction nor in vitro ATP-dependent actin-myosin sliding was affected by a monoclonal antibody to the 50K–20K junctional peptide in muscle [6], though the antibody (IgG) completely covers actin-binding sites adjacent to the junctional peptide [7], indicating that during muscle contraction, myosin heads do not pass through rigor A-M configuration, determined from extracted protein samples. In addition, it has long been known that X-ray diffraction pattern from contracting muscle is intermediate between those from relaxed muscle and those from rigor muscle, despite a considerable fraction of myosin heads are believed to form rigor A-M complex [8].

To obtain information about the state of myosin heads at the end of power stroke during Ca^2+^-activated contraction in skinned skeletal muscle fibers, we first made the fibers to contract maximally in contracting solution (pCa,4), and then transferred them into high-Ca rigor solution (pCa,4). As soon as the high-rigor state was established, we applied ramp-shaped releases (amplitude, 0.5% Lo; duration, 5 ms) to the high-Ca rigor fibers with the following results [9]: (1) After the initial drop in rigor tension coincident with the applied release, the fibers were found to exhibit slow tension recovery, the time course of which resembled tension recovery of tetanized muscle fibers following applied quick release [10]; (2) the tension recovery is observed for many minutes by repeated application of release-stretch cycles; and (3) the tension recovery disappeared in the presence of EDTA, recovery following ramp-shaped releases. This finding can be interpreted to mean that the tension recovery is produced by A-M-ADP myosin heads [9].

The present experiments were undertaken to give information about the structural changes in the rigor actin-myosin head linkages producing the tension recovery following ramp-shaped releases, by recording the X-ray diffraction pattern from skinned muscle fibers in high-Ca rigor state. These results suggest that (1) during the initial drop in rigor tension coincident with the applied release, the oval-shaped myosin head catalytic domain (CAD) tilts in such a way that its angle of attachment to actin filament changes from oblique to more perpendicular configuration, and (2) after the CAD tilting localized structural changes take place in the rigor actin-myosin head interface, which show up as the tension recovery.

## 2. Results

### 2.1. Effect of Ramp-Shaped Releases on the Equatorial Reflections of High-Ca Rigor Muscle Fibers

**Figure 1:**
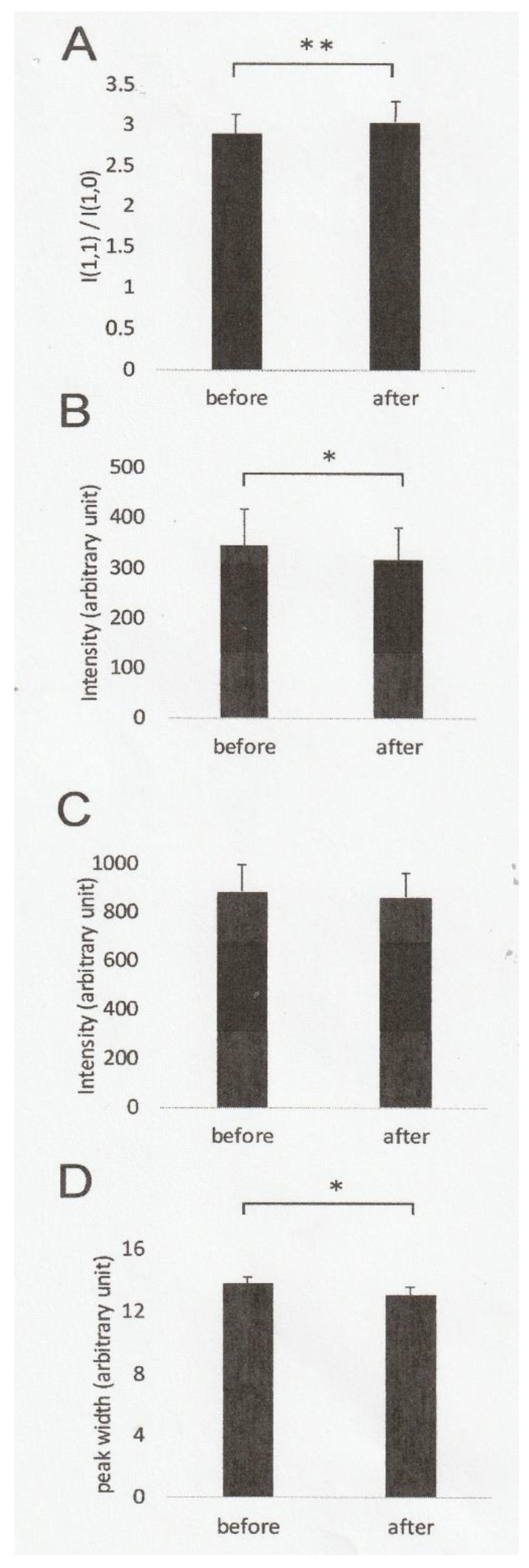
Changes in the equatorial reflections from high-Ca rigor fibers before and after ramp-shaped releases. (**A**) Mean values of the Intensity ratio, I (1,1)/I (1,0), obtained before (left) and after (right) application of ramp-shaped releases. (**B**) Mean values of the 1,1 reflection intensity, obtained before (left) and after (right) ramp-shaped releases. (**C**) Mean values of the 1,1 reflection intensity, obtained before (left) and after (right) application of ramp-shaped releases. (**D**) Mean values of half-width of the 1,0 reflection, obtained before (left) and after (right) ramp-shaped releases. In A–D, vertical bars indicate SEM, while horizontal bars with * and ** indicate significant differences with *p* < 0.05 and *p* < 0.01 (*t*-test) between the corresponding mean values, respectively.

In the present study, the intensity ratio of the two equatorial reflections, I(1,1)/I(1,0), in high-Ca rigor fibers was 2.54 ± 0.23 (mean ± SEM, *n* = 8) before ramp-shaped releases, and was observed to further increase to 2.72 ± 0.30 (*n* = 8) after ramp-shaped releases, indicating that ramp-shaped releases caused an increase in the intensity ratio I(1,1)/I(1,0)(*t*-test, *p* < 0.01) (Figure 1A). The above increase in the intensity ratio resulted from a significant decrease in the intensity of 1,0 equatorial reflection after ramp-shaped releases (p < 0.05, *n* = 8) (Figure 1B), while the intensity of 1,1 equatorial did not change significantly (Figure 1C). The (1,0) lattice spacing, obtained from the distance between the two peaks of 1,0 reflection, was 38.98 ± 0.25 nm (mean ± SEM, *n* = 8) before ramp-shaped releases, and 39.29 ± 0.22 nm (*n* = 8) after ramp-shaped releases, respectively. This result indicates that ramp-shaped releases increases the (1,0) lattice spacing significantly by 0.31 nm (*t*-test, *p* < 0.001). Meanwhile, the decrease in the (1,0) intensity was accompanied by a decrease in the half-width of 1,0 reflection (Figure 1D).

### 2.2. Effect of Ramp-shaped Releases on the Intensity Profile of 14.3 nm Meridional Reflection of High-Ca Rigor Muscle Fibers

**Figure 2:**
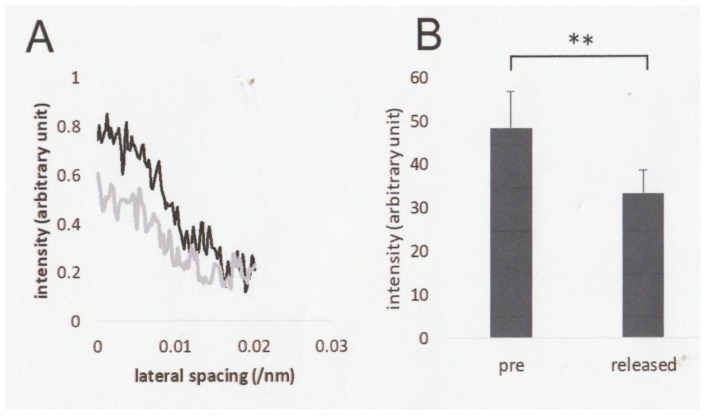
(**A**) Intensity profiles of the 14.3nm meridional reflection before (black line) and after (grey line) ramp-shaped releases. (**B**) Mean intensity values of the 14.3 meridional reflection before (left) and after (right) ramp-shaped releases. Vertical bars indicate SEM (*n* = 8), while horizontal bar with ** indicates significant difference (*p* < 0.01) between the two mean values.

Figure 2A shows intensity profiles of the 14.3 nm meridional reflection, arising from periodic protrusion of myosin heads around myosin filaments [8], before and after ramp-shaped releases. The intensity of the 14.3 nm meridional reflection exhibited a definite decrease after ramp-shaped releases over the whole range of reflection including its peak. The mean integrated intensity of meridional 14.3 nm reflection (arbitrary units) was 43.4 ± 8.5 and 33.4 ± 5.1 (mean ± SEM, *n* = 8) before and after ramp-shaped releases, respectively (Figure 2B).

### 2.3. Effect of Ramp-Shaped Releases on the Intensity Profile of 5.9nm Actin-Based Layer line in high-Ca Rigor Muscle Fibers

**Figure 3:**
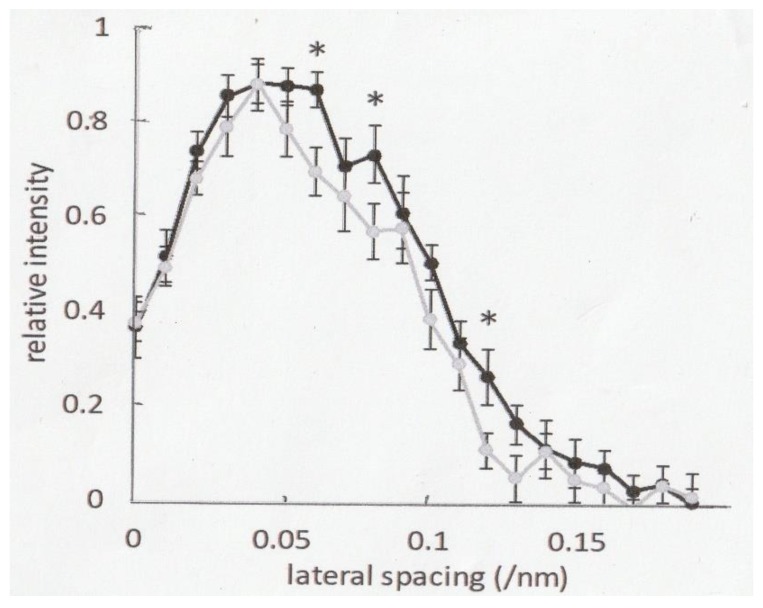
Changes in the average intensity profile of the 5.9 nm actin-based layer lines from high-Ca rigor fibers, obtained from 8 preparations before (black line) and after (grey line) ramp-shaped releases. The intensity profiles were constructed from averaged data points, measured at 20 different positions of lateral spacing. Each asterisk (*) indicates significant difference (*p* < 0.05) between the values before and after release. For further explanation, see text.

As shown in Figure 3, the intensity profile of the 5.9 nm actin-based layer line was found to differ before (thick line) and after (thin line) ramp-shaped releases. The layer line intensities (relative value), measured at 20 points along the reciprocal spacing axis before and after ramp-shaped releases, and their mean values are shown as data points on the thick and thin lines. Bars with * indicate the intensities of lateral spacings, which are significantly smaller after ramp-shaped releases (*p* < 0.05, n = 8).

## 3. Discussion

### 3.1. Tilting of Rigor Myosin Head CAD to Take up Ramp-Shaped Releases

The equatorial X-ray diffraction pattern from vertebrate skeletal muscle arises from the hexagonal lattice of actin and myosin filaments, and has been studied to obtain information about the performance of myosin heads during contraction. During isometric tension development, the intensity of (1,0) reflection decreases, while that of (1,1) reflection increases [11,12,13]. As the 1,0 plane consists only of myosin filaments, while the 1,1 plane consists of both myosin and actin filaments at an ration of 1:2, the above 1,0 and 1,1 intensity changes are accounted for as being due to the movement of X-ray scattering mass, i.e., myosin heads, from the 1,0 to the 1,1 plane [8]. In contracting muscle, myosin heads repeat attachment to, and detachment from, actin filaments, whereas myosin heads remain attached to actin filaments in rigor muscle. Consequently, the intensity ratio, I(1,1)/I (1,0) is larger in rigor muscle than in contracting muscle [8,13].

In the present study, the fibers were in the state of high-Ca rigor, in which all myosin head catalytic domain, which hereafter be abbreviated as CAD, formed tight rigor linkages with actin filaments. We have already reported that quick releases (amplitude, 1% of Lo; duration, ~1 ms), applied to drop Ca-activated isometric tension to zero, give irreversible damage to rigor fibers [9]. This may be taken to indicate that the conventional quick releases (amplitude, 1% of Lo or ~10 nm/half sarcomere; duration, 1 ms) are too large in amplitude (and in velocity) for each rigor linkages to take up applied displacement by changing their attachment angle to actin filaments; as a result, the conventional quick release may cause irreversible damage to myofilament structures, while rigor linkages remain attached. In accordance with this view, it has been reported that when rigor fibers are stretched by <5%, both actin and myosin filament out of the myofilament overlap region exhibit marked irreversible elongation, while the width of the myofilament overlap region remains unchanged [14].

To study mechanical response of rigor fibers without giving damage to muscle fibers, we applied ramp-shaped releases, which was much smaller in amplitude and much longer in duration (amplitude, 0.5% of Lo; duration, 5ms), and succeeded in obtaining reproducible results to characterize mechanical responses of high-Ca fibers to the applied release. A most prominent result obtained was that the tension recovery after ramp-shaped releases could be seen in high-Ca rigor fibers, but not in low-Ca rigor fibers [9]. In the fibers put into high-Ca rigor state by removing external ATP during Ca^2+^-activated isometric force generation, each myosin head is expected to form rigor linkages with actin after performing its last power stroke, thus preserving the myosin head at the end of power stroke. When ramp-shaped releases are applied to high-Ca rigor fibers, the applied displacement (5–6 nm/half sarcomere) would be taken up partly by changes in the attachment angle to actin filament, i.e., tilting, of myosin head in the direction of applied release, and partly by other elastic structures in each sarcomere.

**Figure 4:**
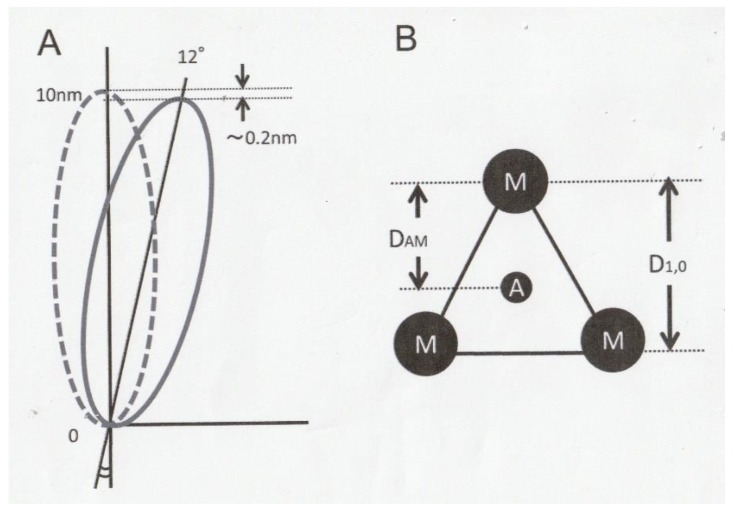
(**A**) Diagram illustrating tilting of myosin head (M) in response to ramp-shaped releases. Ramp-shaped releases are applied to the fiber in the direction from right to left, so that each myosin head CAD (10nm long) tilts around the base, i.e., the proximal end of myosin head CAD (0) from right (solid line) to left (broken line) around the proximal end of myosin head CAD (0). A fraction of the applied displacement (~2 nm out of 5–6 nm/half sarcomere) is assumed to be taken up by tilting of each rigor myosin head CAD (long axis, 10nm) by 12°, from oblique (solid line) to perpendicular (broken line) configuration to actin and myosin filaments. Please note that after a release, the lateral distance between actin and myosin filaments, i.e., D_A-M_, increases by ~0.2nm. (**B**) Schematic diagram showing unit cell of myofilament lattice, in which an actin filament (A) is in trigonal position in an equilateral triangle formed by three myosin filaments (M).

As illustrated in Figure 4, the present results on the equatorial reflections can be accounted for at least qualitatively by assuming that (1) the length of long axis of globular-shaped myosin head CAD is 10 nm [15]; (2) In high-Ca rigor fibers, each myosin head are kept attached to actin filament obliquely in the direction of power stroke, so that the angle of attachment of myosin head CAD, measured from the plane perpendicular to the filament axis, is 12°. (3) On application of ramp-shaped releases, each myosin head CAD takes up a fraction of the applied displacement (~2 nm out of 5-6nm/half sarcomere) by tilting in the direction opposite to that of power stroke. As the result, the angle of attachment of myosin head CAD changes by 12° to make the actin-attached myosin CAD from oblique to perpendicular configuration to actin filaments. The rest of the applied displacement is assumed to be taken up by other elastic structures in each sarcomere.

The above tilting of myosin head CAD increases the lateral distance between actin and myosin filaments, D_AM_, by 0.21 nm [10 − (10cos12°)] (Figure 4A). Considering the unit cell of actin and myosin filament lattice, in which each actin filament is located in trigonal position [8], the 1,0 myosin filament lattice spacing, D_M-M_, is related to D_A-M_, as D_M-M_ = √3 cos30°D_A-M_ = 1.5D_A-M_ (Figure 4B). Therefore, an increase in D_A-M_ by 0.21 nm results in an increase in D_M-M_ by 0.33 nm, a value practically equal to the amount of increase in D_M-M_, 0.31 nm, after ramp-shaped releases observed in the present study. Meanwhile, I (1,0) showed a definite decrease after ramp-shaped releases (*t*-test, *p* < 0.05). This result might be due to some disorder of myosin filament lattice structure, caused by elastic recoil of elastic structures in between myosin heads CAD after ramp-shaped releases.

### 3.2. Helical Rotation of Rigor Myosin Heads As a Possible Cause of the Tension Recovery of High-Ca Rigor Muscle Fibers After Ramp-Shaped Releases

The 5.9 nm actin layer line results from the double helical structure of actin filaments with a subunit pitch of 5.9 nm. Its intensity increases markedly during muscle contraction, indicating attachment of myosin head CAD to actin filaments [8]. In our previous studies, we have shown that (1) the tension recovery following ramp-shaped releases can also be observed in the fibers put into rigor state by removing external ATP from low-Ca rigor solution(pCa, >9), and (2) EDTA, chelating Mg ions, eliminates the tension recovery in low-Ca rigor fibers, but has no effect on the tension recovery in high-Ca rigor fibers [9], suggesting that it is A-M-ADP myosin heads that causes the tension recovery. The time course of tension recovery following ramp-shaped releases in high-Ca rigor fibers resembles that of quick tension recovery following quick releases in tetanized muscle fibers [10], though the time scale is three order of magnitude slower in the former than in the latter [9]. In the present study, the intensity profile of the 5.9 nm actin-based layer line was observed to change after ramp-shaped releases, in such a way that the intensity decreased significantly at the descending limb of the intensity profile (Figure 3). The 5.9 nm actin-based layer lines are generally believed to result from helix of actin subunits that has a pitch of ~5.9 nm with an axial translation of ~2.75 nm [8]. Considering the results shown in Figure 3, some changes take place in the vicinity of actin-myosin head interface. Although we cannot at present clearly explain the results on the intensity changes of 5.9 nm layer line, it seems likely that after ramp-shaped releases, myosin head slightly rotates along the helical strand of actin filaments, to result in a decrease of the 5.9 nm layer line intensities observed in the present study. This may be taken to imply that the tension recovery may result from weakening of rigor linkages between the A-M-ADP myosin heads and actin filaments, from the tight static condition to a less tight flexible condition. Considering the extremely slow time course of tension recovery, which is complete in >30 s [9], the helical rotation of rigor A-M-ADP myosin heads is an extremely slow process, due to the static nature of rigor muscle fibers. In this connection, it seems possible that muscle tension generation in contracting muscle also results from helical motion of A-M-ADP myosin heads around the helical structure of actin filaments. In this connection, it is of interest that Jarosch suggests actin filament rotation as a possible cause of muscle force generation [16,17]. It is also of interest that recent development of cryo electron microscopy reveals the Ca^2+^-dependent structural changes of actin filaments, with regulatory proteins attached [18]. It is in our future planning to study the role of actin filaments as well as myosin heads in mechanisms of muscle force generation, which are of paramount importance not only in the field of muscle physiology but also in the field of exercise physiology and rehabilitation medicine.

Though not directly related to the present study, it should be noted that several papers have been published on the mechanical responses of rigor muscle fibers [19,20,21], and on the biochemical characteristics of skinned fibers, myofibrils and extracted myosin fragments to obtain information about possible functional interactions between the two myosin heads in a myosin molecule [22,23,24]. It is also our future program to give answer to the old, fundamental question, ”why does myosin have two heads?”

## 4. Materials and Methods

### 4.1. Preparation

White male rabbits weighing (Japan White, Sankyo Lab. Industry, Tokyo, Japan) were killed by sodium pentobarbital injection (50 mg/kg) into ear vein, and psoas muscles were dissected from them. The rabbits were treated following the Guiding Principles for the Care and Use of Animals in the Field of Physiological Sciences, published by the Physiological Society of Japan. The protocol was approved by the Teikyo University Animal Care Committee (protocol #07-050, 15 September 2012). The method of preparing strips of chemically skinned muscle fibers has been described by Sugi et al. [9]. Single muscle fibers (diameter, 40–60 μm) were isolated from the fiber strips, and mounted horizontally in an experimental chamber (volume, 1 mL) between a force transducer (Kulites Semiconductor Products, Inc, Leonia, NJ, USA) and a servomotor (MP12, Mitsubishi Materials) by tying both ends with silk threads [9]. Other details of the method have been described elsewhere [9].

### 4.2. Solutions

Relaxing solution (pCa,>9) contained 120 mM KCl, 5 mM MgCl_2_, 4 mM ATP, 10 mM EGTA, and 20 mM PIPES. Contracting solution (pCa,4) was prepared by adding 4mM CaCl_2_ to maximally activate the fiber. High-Ca rigor solution (pCa,4) was prepared by removing ATP from contracting solution. The pH of all solutions was adjusted to be 7.0 with PIPES. In some cases, hexokinase (50 unit/mL) and D-glucose (2 mM) were added to facilitate removal of ATP in the experimental solution with similar results, indicating that the present results may not be influenced by ATP or ADP contaminations. Exchange of solutions was made by first draining a solution in the chamber, and then refilling the chamber with another solution.

### 4.3. Recording of X-ray Diffraction Pattern from Rigor Fibers Before and After Ramp-Shaped Releases

X-ray diffraction patterns were recorded from small bundles consisting of 2–3 skinned muscle fibers. The fiber bundles were mounted horizontally between the servomotor and the force transducer. Diffraction images were recorded using an imaging plate system (BAS 2500, Fuji Xerox, Tokyo, Japan). The wavelength and the size of X-ray beam was 0.09 nm and size, 250 × 150 μm, respectively, and the specimen-to-detector distance was 1.8 m. The fiber was kept at its slack length Lo (~3 mm) at a sarcomere length of 2.0–2.4 μm, which was measured by optical diffraction by He-Ne laser light [9]. Dithiothreitol (5 mM) and catalase (1000 U/mL) were added to the experimental solution to prevent damage of the fibers from free radicals produced by X-rays [25].

### 4.4. General Procedure

**Figure 5:**
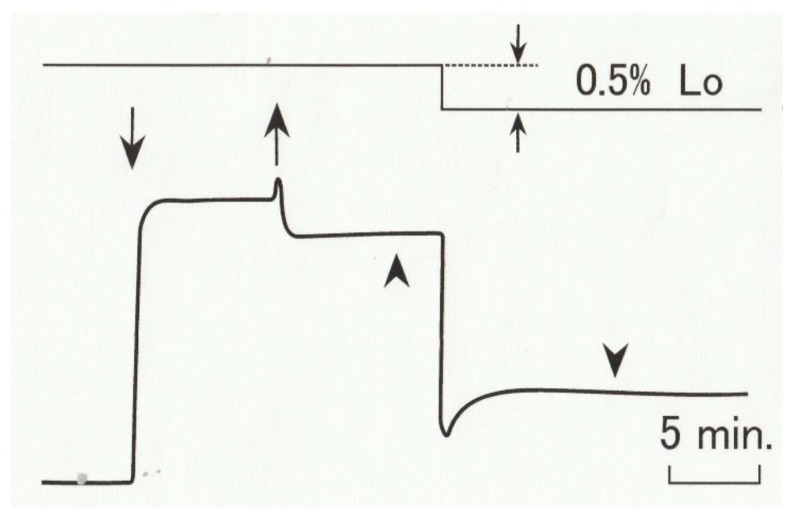
Diagram illustrating the time course of tension changes in high-Ca rigor muscle fibers in response to a ramp-shaped release. The fibers, kept in relaxing solution, are put into contracting solution (pCa, 4), and after full isometric tension was developed transferred to high-Ca rigor solution (pCa, 4). After high-Ca rigor state is established, the fibers were subject to a ramp-shaped release. The time of application of contracting and high-Ca rigor solutions are indicated by arrows, while the fiber length changes are shown above the tension record. Arrowheads indicate times of recording of X-ray diffraction pattern from rigor fibers before and after a ramp-shaped release.

The fibers were first kept in an acrylate experimental chamber (volume, 1 mL) filled with relaxing solution, and then transferred them to contracting solution. As soon as the maximum Ca^2+^-activated tension was developed, the fibers were further transferred to high-Ca rigor solution to put them into high-Ca rigor state. The time of establishment of rigor state after application of rigor solution was estimated to be <20 s [9]. Ramp-shaped releases (amplitude, 0.5% of Lo, or ~6 nm/half sarcomere) were applied to rigor fibers with the servomotor, while the tension changes were recorded with the force transducer [9]. As shown in Figure 5, the initial drop in rigor tension coincident with the applied release was followed by a small but distinct tension recovery towards a steady level. To obtain information about structural changes in actin and myosin filaments producing the tension recovery following the release, X-ray diffraction patterns of the fibers was recorded (1) ~3 min before, and (2) ~10 min after the application of releases, i.e., (1) during the steady rigor tension generation, and (2) during the steady rigor tension following the tension recovery. The exposure time of the fibers to X-rays was 10 s. The experiments were made at 15 °C.

### 4.5. Data Analysis

**Figure 6:**
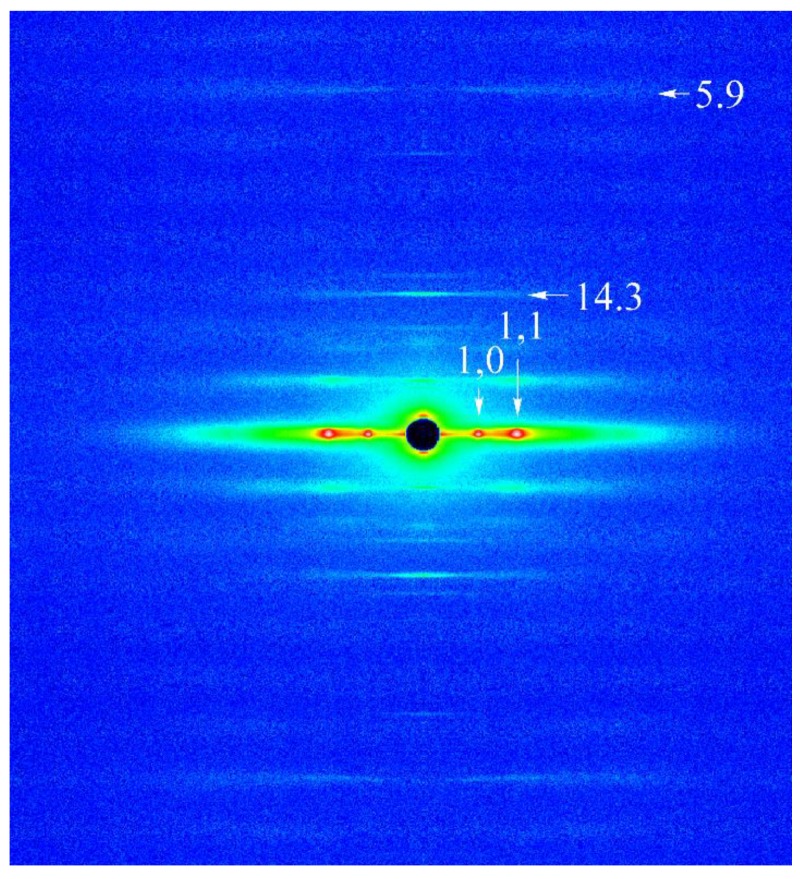
2D X-ray diffraction patterns from high-Ca-rigor fibers, obtained before ramp-shaped releases. Equatorial (1,0) and (1,1) reflections, meridional 14.3 nm reflection, and 5.9 nm actin-based layer lines are indicated by vertical and horizontal arrows, respectively.

Figure 6 shows a typical 2D X-ray diffraction pattern from high-Ca rigor muscle fibers, obtained before ramp-shaped releases. Integrated intensities of the 1,0 and the 1,1 equatorial reflections were obtained by measuring the area under the peaks of diffraction pattern, after subtracting background intensity [26]. The values of 1,0 lattice spacing were obtained by measuring the distance between the two peaks of 1,0 reflection. The intensity profile of 14.3 nm meridional reflection, arising from helical periodic protrusion of myosin heads around myosin filament, was obtained by scanning it along the equatorial axis. The intensity profile of actin-based 5.9 nm layer lines at both sides of the meridian was obtained by scanning them along the equatorial axis.
